# Multiple Loops of External and Internal Carotid Arteries Vulnerable in Surgical and Radiological Procedures

**DOI:** 10.4274/balkanmedj.2017.1179

**Published:** 2018-05-29

**Authors:** Satheesha Nayak, Naveen Kumar

**Affiliations:** 1 Department of Anatomy, Melaka Manipal Medical College, Manipal Campus, Manipal Academy of Higher Education, Manipal, Karnataka, India

Carotid arterial system tortuosity is comparatively less common than its variations in branching patterns and termination. Tortuous common carotid artery is often associated with risk of injury during surgical procedures in the neck region, and such injuries usually occur during tracheotomy (1). Looping or tortuosity of internal carotid arteries can also be observed with advancing age (2). However, remarkable external carotid artery tortuosity is also risky during surgeries conducted in the cervical region. In the present case, we report a unilateral occurrence of multiple loops along the length of both the external carotid artery and internal carotid arteries on the left side of the neck of an adult male cadaver, aged approximately 70 years, donated to the department for educational and research purposes (Figure 1, 2). The vessels on the right side did not show any looping along their length. The common carotid artery was straight and divided into two terminal branches at the level of the upper border of the lamina of the thyroid cartilage. The external carotid artery showed three characteristic loops before its termination. The uppermost loop, before the artery entered the parotid gland, was the largest. Its branching pattern and termination were unremarkable. The internal carotid arteries was ‘S’-shaped because of two loops: one near its origin, and the other at its entry point into the carotid canal. The loop near the carotid canal was horizontally placed and laterally directed. Arterial tortuosity may result in arterial kinking, and its occlusion may lead to an ischemic attack. A tortuous common carotid artery course could also cause dysphagia lusoria, which is uncommon in elder individuals with hypertensive and atherosclerotic lesions (3). It has also been suggested that the presence of tortuous common carotid artery may mimic neck region aneurysms, and in such cases, performing arteriography is contraindicated and carries a risk of arterial injury (4). A tortuous internal carotid arteries course is known to cause various adverse effects, including syncope, blackout, dizziness, and vertigo; it could also result in episodes of cerebrovascular insufficiency related to head positions (5). To reduce the risk of operative morbidity and mortality in patients undergoing surgery, knowledge of carotid arterial loops is essential for various surgical approaches performed in the head and neck regions. There is a very high probability of injuring one of the five loops found in the current case during skull base surgery, maxilla-facial and cervical lymph node clearance surgery, and in some radiological procedures.

The occurrence of multiple loops of carotid vessels presented in this case is unique and has not yet been reported. These loops may decrease blood flow to certain regions of the head and neck, when in certain positions. Knowledge of these unusual loops may be important in diagnostic, therapeutic, radiological, and surgical techniques.

## Figures and Tables

**Figure 1:**
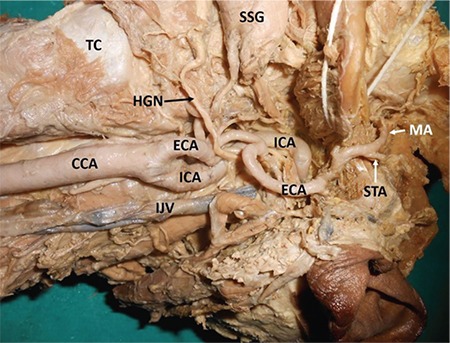
Left carotid vessels showing multiple loops. *CCA: common carotid artery; ECA: external carotid artery; HGN: hypoglossal nerve; ICA: internal carotid artery; IJV: internal jugular vein; MA: maxillary artery; SSG: submandibular salivary gland; STA: superficial temporal artery; TC: thyroid cartilage*

**Figure 2:**
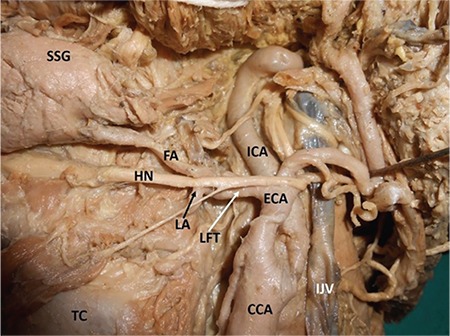
Closer view of multiple loops of left carotid vessels. *CCA: common carotid artery; ECA: external carotid artery; FA: facial artery; HN: hypoglossal nerve; ICA: internal carotid artery; IJV: internal jugular vein; LA: lingual artery; LFT: linguofacial trunk; SSG: submandibular salivary gland; TC: thyroid cartilage*
